# Supplementary material for: Different Requirements of CBFB and RUNX2 in Skeletal Development among Calvaria, Limbs, Vertebrae and Ribs
Source: Int J Mol Sci. 2022 Oct 31;23(21):13299. doi: 10.3390/ijms232113299 (PMC9657020; doi:10.3390/ijms232113299)
Supplement: Supplementary file 1 [file ijms-23-13299-s001.zip › ijms-1874284-supplementary.pdf]

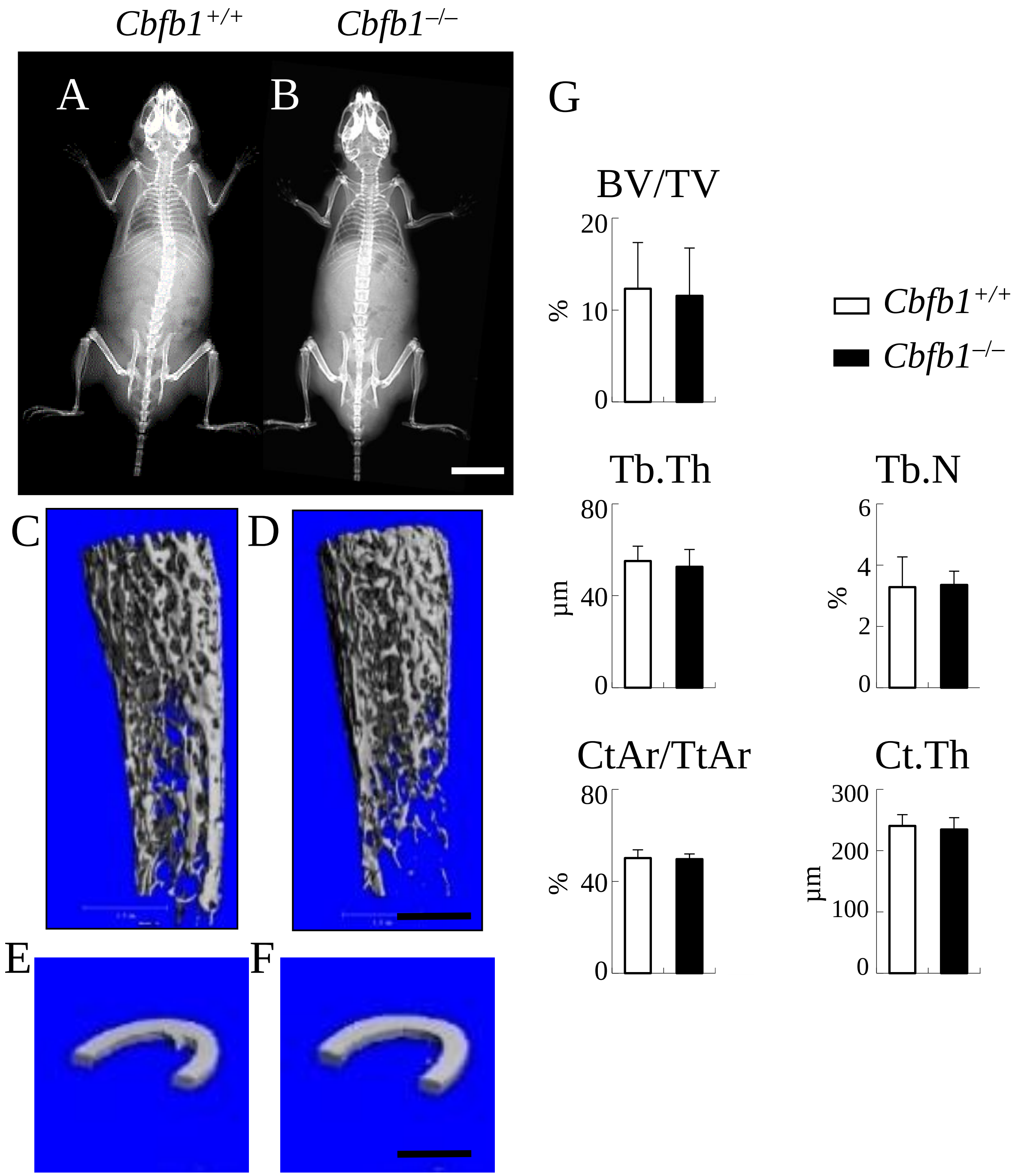

Supplementary Figure S1.

Micro–CT analyses of femurs in *Cbfb1*$^{+/+}$ and *Cbfb1*$^{-/-}$ mice at 10 weeks of age. (A, B) X-ray analysis at 10 weeks of age. (C–F) Three-dimensional trabecular bone architecture of distal femoral metaphysis (C, D) and cortical bone at mid–diaphysis (E, F) in femurs. Scale bars: 1cm (A, B) and 1mm (C–F). (G) Quantification of the trabecular bone volume (bone volume/tissue volume, BV/TV), trabecular thickness (Tb.Th), trabecular number (Tb.N), the cortical area (CtAr/TtAr), and cortical thickness (Ct.Th). Seven *Cbfb1*$^{+/+}$ and *Cbfb1*$^{-/-}$ mice were analyzed.

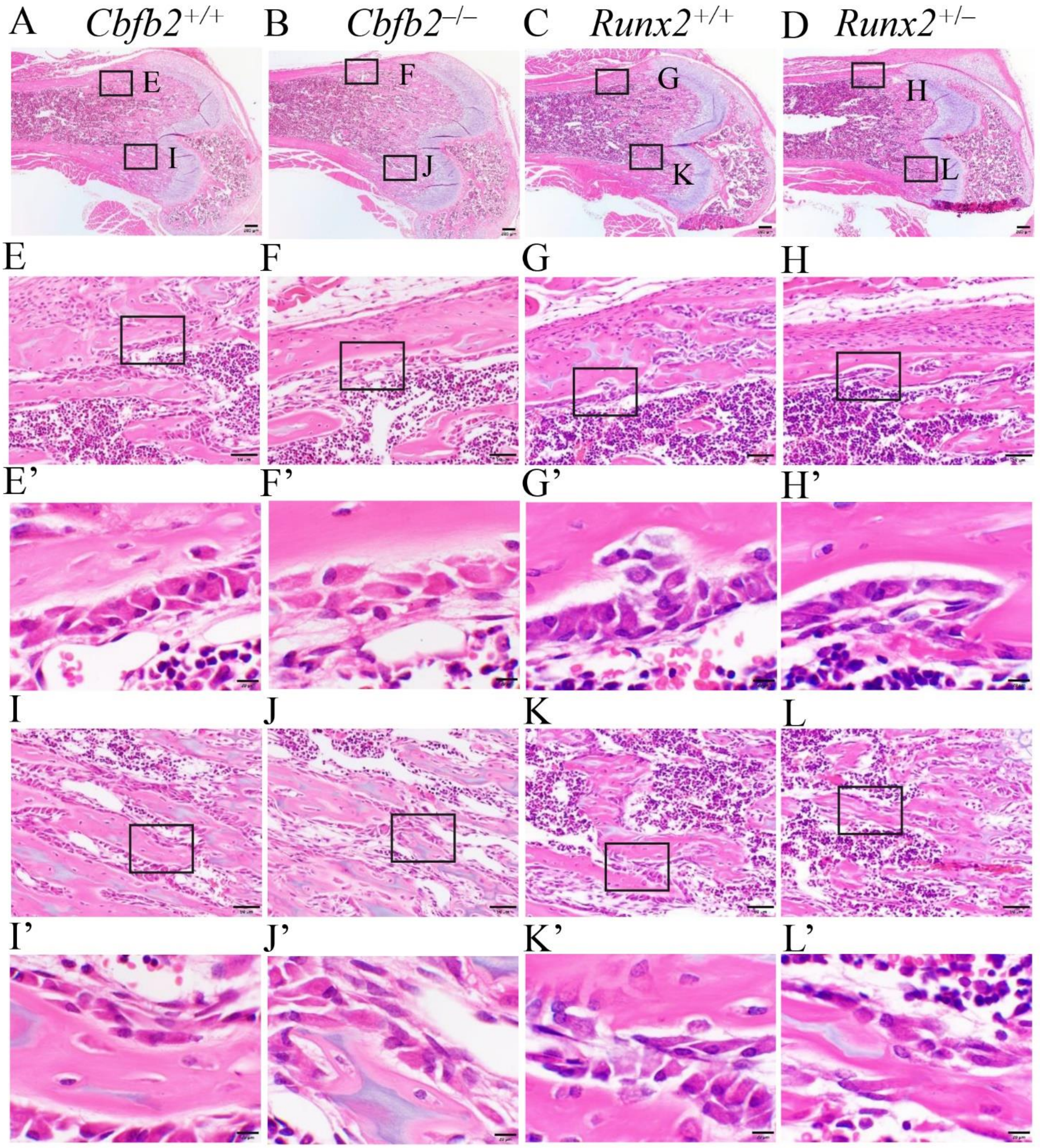

Supplementary Figure S2.

Histological analyses of femurs at 4 weeks of age. H-E staining of femoral sections in *Cbfb2*$^{+/+}$ (A, E, I), *Cbfb2*$^{–/–}$ (B, F, J), *Runx2*$^{+/+}$ (C, G, K) and *Runx2*$^{+/–}$ (D, H, L) mice at 4 weeks of age. The boxed regions in A–D are magnified in E and I, F and J, G and K, and H and L, respectively. The boxed regions in E–L are magnified in E′–L′, respectively. Scale bars: 200 μm (A–D), 50 μm (E–L), and 20 μm (E′–L′). The number of mice analyzed: *Cbfb2*$^{+/+}$, n = 3; *Cbfb2*$^{–/–}$, n = 3; *Runx2*$^{+/+}$, n = 3; *Runx2*$^{+/–}$, n = 4.

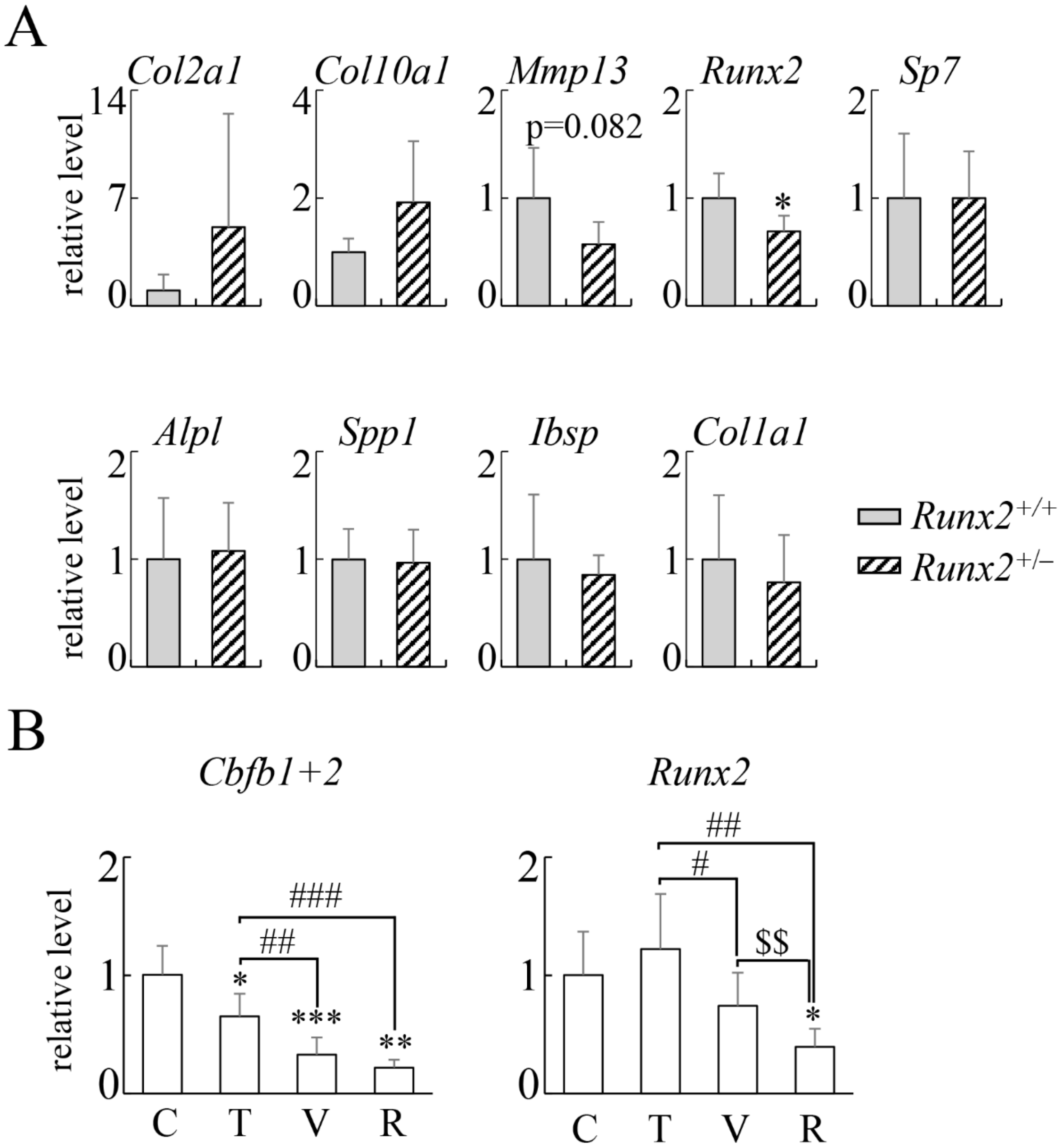

Supplementary Figure S3.

(A) Real–time RT–PCR analysis using rib RNA from *Runx2*$^{+/+}$ and *Runx2*$^{+/-}$ mice at 4 weeks of age. The values of *Runx2*$^{+/+}$ mice were defined as 1 and relative levels are shown. The number of mice analyzed: *Runx2*$^{+/+}$, n = 8; *Runx2*$^{+/-}$, n = 7. (B) Real-time RT-PCR analysis using RNA from calvarias (C), tibia (T), vertebrae (V) and ribs (R) from wild type mice at 4 weeks of age. The values of calvaria were defined as 1 and relative levels are shown. The number of mice analyzed: C, n = 4; T, n = 8; V, n=7; R, n=8. Data are shown as the mean ± SD. *, #p < 0.05, **,##,$$p < 0.01, and ***,###p < 0.001.

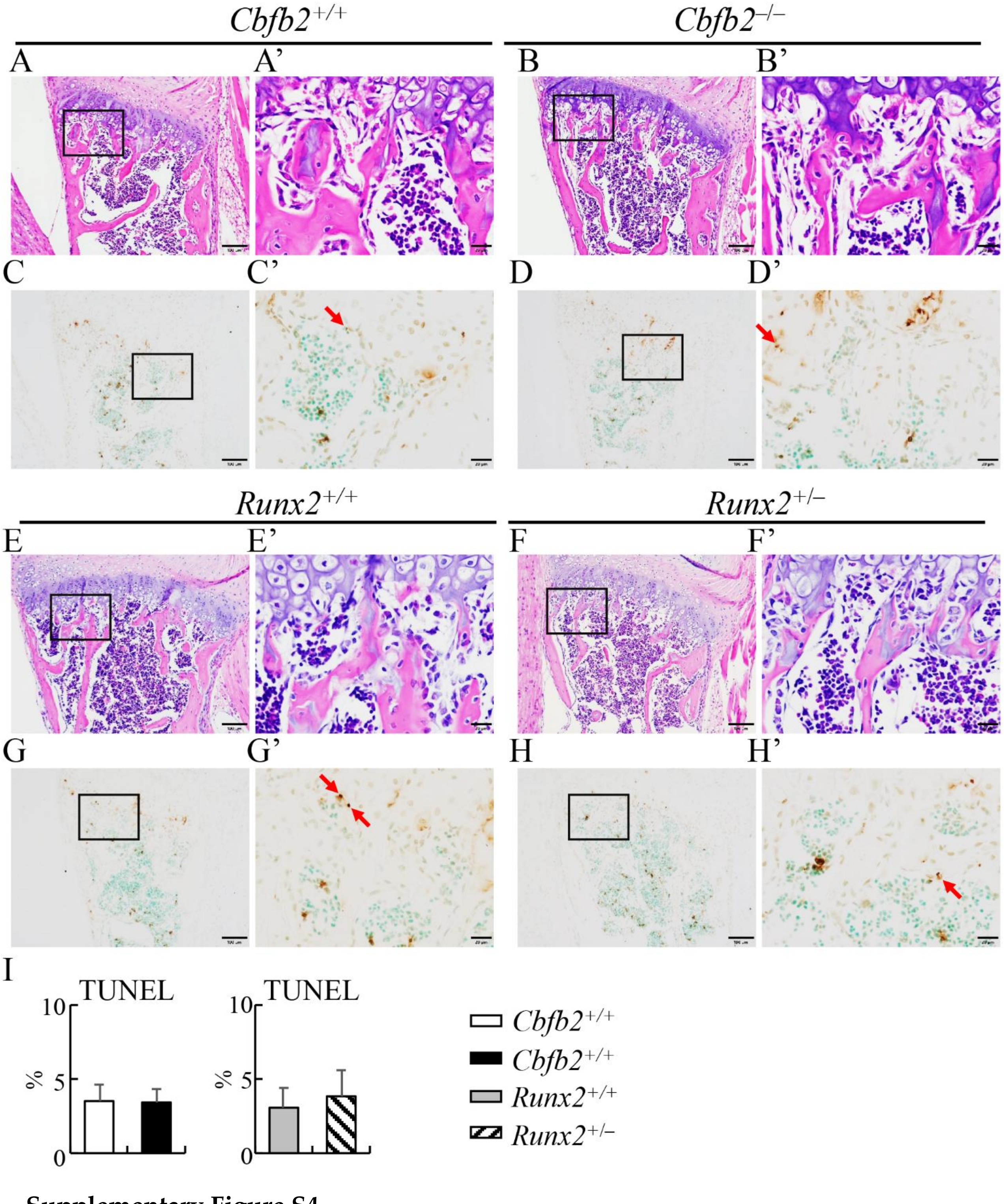

Supplementary Figure S4.

(A–H) H-E staining (A, B, E, F) and TUNEL staining (C, D, G, H) of the sections of 1st lumbar vertebrae in *Cbfb2*$^{+/+}$ (A, C), *Cbfb2*$^{-/-}$ (B, D), *Runx2*$^{+/+}$ (E, G) and *Runx2*$^{+/-}$ (F, H) mice at 4 weeks of age. The boxed regions in A–H are magnified in A′–H′, respectively. Scale bars: 100 μm (A–H) and 20 μm (A′–H′). (I) The frequencies of TUNEL–positive osteoblast-like and osteoprogenitor-like cells shown by arrows were counted in C′, D′, G′ and H′. Data are shown as the mean ± SD. The number of mice analyzed: *Cbfb2*$^{+/+}$, n = 3; *Cbfb2*$^{-/-}$, n = 3; *Runx2*$^{+/+}$, n = 3; *Runx2*$^{+/-}$, n = 4. Two regions were counted in each mouse.

age	*Cbfb2*$^{+/+}$ number (%)	*Cbfb2*$^{+/-}$ number (%)	*Cbfb2*$^{-/-}$ number (%)
E15.5-E18.5	148（25）	297（51）	137（24）
P0-P2	114（27）	215（52）	87（21）
4w	87（29）	182（60）	32（11）

Supplementary Table S1.

The numbers and percentages of live *Cbfb2*$^{+/+}$, *Cbfb2*$^{+/-}$ and *Cbfb2*$^{-/-}$ mice obtained in the crossing of *Cbfb2*$^{+/-}$ littermates during the embryonic stage (E15.5-E18.5), newborn stage (P0-P2), and at 4 weeks of age.

Supplementary Table S2.

Primer sequences for real-time RT-PCR :

	Forward	Reverse
	5'------------------------------------3'	5'--------------------------------------3'
Actb	CCACCCGCGAGCACAGCTTC	TTGTCGACGACCAGCGCAGC
Col2a1	ATCTGGTTTGGAGAGACCAT	CTCTACATCATTGGAGCCCT
Col10a1	ATATGCTGCCTCAAATACCC	CTCTTATGGCGTATGGGAT
Mmp13	CTTCTGGCACACGCTTTTCC	ATGGGAAACATCAGGGCTCC
Runx2	AACAAGACCCTGCCCGTG	TGAAACTCTTGCCTCGTCCG
Sp7	AGGCACAAAGAAGCCATAC	AATGAGTGAGGGAAGGGT
Alpl	CGCACGCGATGCAACACCAC	TGCCCACGGACTTCCCAGCA
Spp1	GCAGAATCTCCTTGCGCCAC	CGAGTCCACAGAATCCTCGC
Ibsp	TGGAGACGGCGATAGTTC	CTAGCTGTTACACCCGAGAG
Col1a1	CCTGGAATGAAGGGACACCG	CCATCGTTACCGCGAGCACC
Bglap&Bglap2	ACTCCGGCGCTACCTTGGAGCC	GCAGGGTTAAGCTCACACTG
Runx1	CGGCCATGAAGAACCAGGTA	TGGTAGGTGGCAACTTGTGG
Runx3	AACCAAGTGGCCAGGTTCAA	TGATGGCTCGGTGGTAGGTA
Cbfb1	GCTCCCATGATTCTGAATGG	TTGCTGTCTTCTTGCCTCCA
Cbfb2	ATCTCCACAGATTGGATGGT	TGCTGTCTTCTTGCCAGTTA
Cbfb1+2	TTAGAGAGAGAAGCAGGCAA	TCTTCTTCGAGCCTCTTCAA
Runx2(Sup)	TCCACCACGCCGCTGTCT	TCAGTGAGGGATGAAATGCT
